# Are immigrants more vulnerable to the socioeconomic impact of COVID-19? A cross-sectional study in Amadora Municipality, Lisbon metropolitan area

**DOI:** 10.3389/fpubh.2022.920308

**Published:** 2022-08-01

**Authors:** Maria Rosario O. Martins, Ahmed Nabil Shaaban, Ana Abecasis, Zelia Muggli, Regina Amado, Dora Vaz, Sara S. Dias, Antonio C. Silva, Ines Fronteira

**Affiliations:** ^1^Global Health and Tropical Medicine (GHTM), Institute of Hygiene and Tropical Medicine (IHMT), NOVA University of Lisbon, Lisbon, Portugal; ^2^Amadora Primary Care Health Centers Group, Regional Health Administration of Lisbon and Tagus Valley, Lisbon, Portugal; ^3^ciTechCare—Center for Innovative Care and Health Technology, Polytechnic of Leiria, Leiria, Portugal; ^4^School of Health Sciences, Polytechnic Institute of Leiria, Leiria, Portugal; ^5^Public Health Department, Regional Health Administration of Lisbon and Tagus Valley, Ministry of Health, Lisbon, Portugal; ^6^AJPAS—Associação de Intervenção Comunitária, Desenvolvimento Social e de Saúde, Amadora, Portugal

**Keywords:** COVID-19, immigrants, socioeconomic impact, inequality, vulnerabilities, Lisbon, Portugal

## Abstract

**Introduction:**

Immigrants carry an extra burden of morbidities and mortalities since the beginning of the coronavirus disease 2019 (COVID-19) pandemic. Pre-existing inequalities among immigrants may threaten their economic wellbeing during the pandemic. This study analyzed the socioeconomic impact of COVID-19 on immigrants and natives living in Amadora, Metropolitan Region of Lisbon and the extent to which preexisting inequalities had been exacerbated during the pandemic.

**Materials and methods:**

This cross-sectional study was conducted in Amadora Municipality, Lisbon Region, through phone interviews and using a structured questionnaire. Data collected in July 2020, included information on a cohort of 420 households, of which 51% were immigrants. To evaluate the socioeconomic position and economic wellbeing changes occurring during the pandemic we estimate crude and adjusted odds ratio (OR) and 95% CI, using Portuguese natives as the reference group.

**Results:**

Overall, 287 (70%) participants responded to the questionnaire, of which 47% are immigrants. Preexisting socioeconomic inequalities were exacerbated during the pandemic. Compared with natives, immigrants were more likely to experience job loss, temporary lay-off, and income loss during the COVID-19 pandemic. Immigrants were also more likely to face several kinds of financial hardship during the pandemic, such as difficulties in buying food, hygiene products, and paying bills.

**Conclusion:**

To the best of our knowledge, this study is the first to capture the direct socioeconomic impact of COVID-19 among immigrants and natives in Portugal. It highlights the bidirectional relation between inequalities deeply rooted among immigrants and COVID-19. Socioeconomic inequalities affect local patterns of COVID-19 burden, as confirmed in previous studies, but COVID-19 also has an impact on the economic wellbeing of Amadora immigrants during the pandemic. Urgent policies must be implemented to mitigate the economic burden of COVID-19 among immigrants, namely in Amadora, Lisbon Region.

## Introduction

Since it was reported in December 2019, the coronavirus disease 2019 (COVID-19) pandemic caused by the severe acute respiratory syndrome coronavirus 2 (SARS-CoV-2) has spread across the globe, causing more than 490 million infections and about 6 million deaths (1). As the disease spread, immigrants have also been substantially affected by the pandemic in what seems to be a disproportional manner. COVID-19 has exacerbated vulnerabilities among immigrants, which were previously caused by long-standing limited access to healthcare, socioeconomic inequalities, and health disparities (2–5). Since the beginning of the pandemic, these preexisting inequalities have translated into higher morbidity and mortality among immigrants in Europe and the United States (5–8). Explanation of how these pre-existing conditions can affect the spread of COVID-19 among immigrants and the importance of universal and equitable access to healthcare services during and after the COVID-19 crisis has already been clarified elsewhere (4).

In Portugal, from 3 March 2020 to 8 April 2022, more than 3.6 million cases of COVID-19 have been notified with 21,851 COVID-19 deaths (9). As a result of the measures undertaken to mitigate the pandemic, such as partial and total lockdown, the unemployment rate was 8.2% in August 2020, compared to only 6% reported in May of the same year (10). In addition, the contraction in the Portuguese economy was 8.4% in 2020 due to COVID-19 (11). Moreover, the country's once-booming tourism sector, which contributed about 15% to Portugal's gross domestic product in 2018, collapsed in 2020 due to coronavirus (12, 13).

Immigrants in Portugal are less likely to access health services compared to natives (14). In an effort to improve immigrants' access to healthcare, Portugal has temporarily regularized all immigrants, including asylum seekers, who have applied for a residence permit before the declaration of the state of emergency on 18 March 2020 (15). Although this step has been appreciated at a regional and international level, it might not be enough to address preexisting conditions that are deeply rooted among immigrants in Portugal, since it is expected that the financial crisis caused by the COVID-19 pandemic will aggravate the economic situation among immigrant's populations. Lessons learned from the previous financial crisis showed that immigrants are among the first groups to be affected (16–20). Previous research shows that the social and economic consequences of the pandemic are hitting disadvantaged groups harder (21). Also, the existing evidence illustrates that COVID-19 has affected people unequally because of pre-existing unequal living and working conditions, as is the case of migrant populations. However, in this review (21), few studies are related to the EU, and no references can be found related to immigrants in Portugal. The objective of this study is to analyze the socioeconomic impact of COVID-19 on immigrants and natives living in Amadora, Metropolitan Region of Lisbon and the extent to which preexisting inequalities had been exacerbated during the pandemic.

## Methods

### Setting

**Figure 1:**
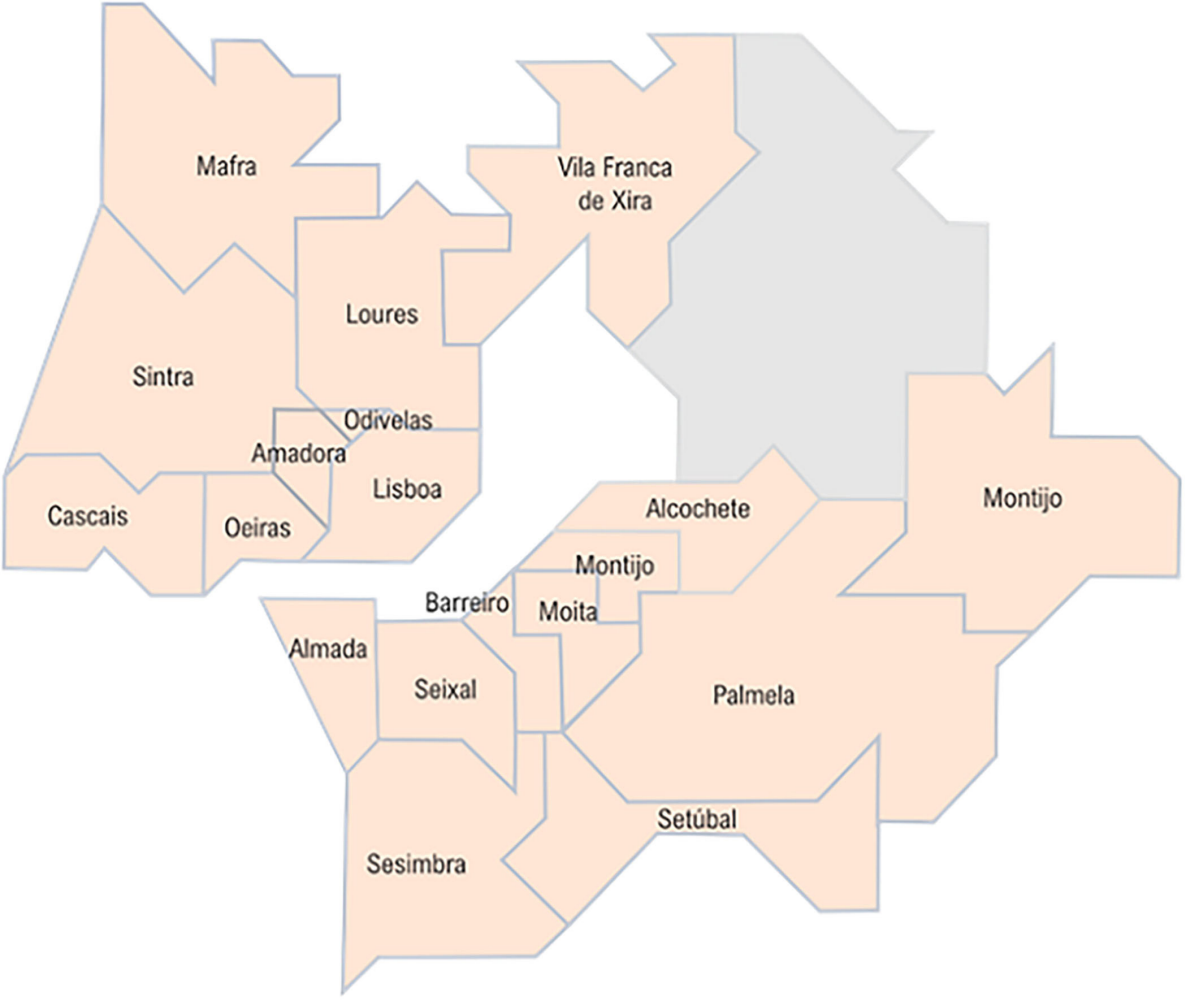
Municipalities in Lisbon metropolitan area. Source: EMTA. https://www.emta.com/spip.php?article1405&lang=en.

Amadora is the most densely populated municipality in the country and the fourth most populous city in Portugal; moreover, 10% of its population has a foreign nationality, namely, from Portuguese-speaking countries (Brazil and Lusophone African countries). Census 2011 data suggest that the socioeconomic conditions of the population living in Amadora Municipality are comparable to those of the Metropolitan Region of Lisbon as a whole, except for the educational level, where Amadora is at a disadvantage. Between June and the beginning of August 2020, Amadora remained among the municipalities in Portugal with a higher incidence of SARS-CoV-2 infection and in a situation of contingency (while the remaining country was in a situation of alert). Due to this situation, and while the country was slowly deconfining, in Amadora some restrictions remained (e.g., shops closing by 8 pm, with exception of supermarkets, petrol stations, clinics, pharmacies, and gyms; restrictions on selling alcohol and limitation on social gathering to 10 persons). A special task force was created to deal with this situation that improved in August. Figure 1 shows the location of Amadora Municipality with the Metropolitan region of Lisbon.

### Design

This study is nested within the prospective cohort study of native and immigrant children and their parents/caregivers living in Amadora, Metropolitan Area of Lisbon, Portugal which started in 2019. In brief, this cohort study collects quantitative data annually coming from 3 different sources: questionnaires (face-to-face and phone interviews), health center administrative data, and hospital records and the whole information is linked in a unique data set. The first data collection corresponded to the baseline assessment and was conducted between June 2019 and the 1st week of March 2020 (before the first case of SARS-CoV-2 infection had been notified in Portugal). At the baseline, we collected information on the socioeconomic and demographic characteristics and migration history of parents/caregivers and children's health outcomes.

Participants were recruited at Amadora health centers during children's consultations. The target population includes all immigrant households with children born in 2015 (starting point of the children's cohort) and the same number of native households with children also born in 2015. Because of the COVID-19 pandemic, we concluded the recruitment 3 months earlier than foreseen, in the 1st week of March 2020, with 420 children/households enrolled with complete data (22). In the present study, the participant is one of the adults living in the household (one of the child's caregivers). At the baseline, about 51% of the 420 adults are immigrants, mainly from Cape Verde, Brazil, Angola, and Guinea-Bissau.

### Participants

For the baseline study (*n* = 420), participants were recruited, between June 2019 and the 1st week of March 2020. Two questionnaires were implemented, face-to-face, to collect data on the main socioeconomic and demographic characteristics of the households and migration history (for immigrants).

In July 2020, 4 months after the notification of the first case in Portugal, and during the Amadora partial lockdown (23), we invited adult participants (*n* = 420) to answer a semistructured pilot-tested questionnaire divided into four sections: changes in employment and household income during the COVID-19 pandemic; changes in material deprivation; difficulties during the lockdown; and difficulties in accessing healthcare. Participants were contacted by phone and in absence of a response, a second and a third call were made.

### Questionnaire variables

The baseline questionnaire, conducted in 2019 by face-to-face interview, explored sociodemographic, economic, and living conditions and when conducted for immigrants (born outside the EU and living in Amadora) also addressed their history of migration. Demographic variables included sex, age (≥18 <35 or ≥35 years), and place of birth. Variables that measure socioeconomic status included employment status: employed, unemployed, and others (domestic, students, retired, and social integration income); family monthly income, originally measured in five categories was dichotomized into <750; ≥750 Euros, where 750 represents the lower limit of the median income class. This option was chosen due to the small number of frequencies in cross tables obtained with the original categories and *n* = 287. Occupations are classified according to the Portuguese Classification of Professions adapted from the International Standard Classification of Occupations (ISCO-08), categorized, for the analysis in: high-skilled occupations (managers; professionals; legislators; technicians; and armed forces occupations), and low-skilled and medium-skilled occupations (personal service workers; industrial workers; unqualified workers; and students). The level of education was measured according to the International Standard Classification of Education (ISCED) adopted by the United Nations Educational, Scientific and Cultural Organization (UNESCO), categorized into 3 classes: professional and higher education, secondary education, and less than secondary education (24).

The questionnaire on the socioeconomic impact of COVID-19 was administrated in July 2020 and included, among others, eight variables related to the impact of COVID-19 on participants' economic wellbeing, defined as follows: unemployed because of COVID-19 (no and yes), on layoff because of COVID-19 (no, yes, and not applicable), change in monthly household income due to COVID-19 (increased or remained the same, decreased), falling behind with bills (no and yes), financial difficulties in buying food (no and yes), financial difficulties in buying hygiene products (no and yes), financial difficulties to pay phone and internet (no and yes), and if kids go to school for a meal when schooling was interrupted during lockdown (no and yes).

### Statistical analysis

Baseline sociodemographic characteristics (categorical variables) across immigrants and native Portuguese were compared using the *x*^2^-test; associations between social inequalities at the baseline were estimated using logistic regression where natives were the reference class. Logistic regression was also used to compute the odds ratio and CI95% of an immigrant being unemployed due to COVID-19, being on lay-off, having household income change, falling behind with bills, or having financial difficulties, when compared to a native, and adjusting for sex, age, education level, employment status, occupation, and family income. Statistical analyses were conducted with STATA^®^, version 13 (Stata Corp LP, College Station, Texas, USA) and figures in R version 4.1.

## Results

The response rate was 70% (*n* = 287) and did not differ by immigrant status (Natives—born in Portugal, Immigrants—born abroad and outside the EU). A total of 152 (53%) participants were natives, whereas 135 (47%) were immigrants. Table 1 presents the main baseline characteristics, collected during 2019 (before the COVID-19 pandemic), according to immigrant status.

**Table 1:** Characteristics of the study sample by immigrant status.

	**All (*****n*** = **287)**		**Natives (*****n*** = **152)**		**Immigrants (*****n*** = **135)**		
	***n***	**(%)**	***n***	**(%)**	***n***	**(%)**	***p*-value**
**Sex (*****n*** **=** **287)**							
Male	32	(11.2)	17	(11.2)	15	(11.1)	
Female	255	(88.8)	135	(88.2)	120	(88.9)	0.984
**Age (*****n*** **=** **287)**							
18–34	138	(48.1)	69	(45.4)	69	(51.1)	
≥35	149	(51.9)	83	(54.6)	66	(48.9)	0.333
**Education (*****n*** **=** **286)**							
Professional and higher education	70	(24.5)	47	(30.9)	23	(17.2)	
Secondary education	106	(37.1)	54	(35.5)	52	(38.8)	
Less than secondary education	110	(38.5)	51	(33.6)	59	(44.0)	0.021
**Employment (*****n*** **=** **287)**							
Employed	212	(73.9)	125	(82.2)	87	(64.4)	
Unemployed and others	75	(26.1)	27	(17.8)	48	(35.6)	0.001
**Occupation (*****n*** **=** **287)**							
High-skilled occupations^a^	202	(70.4)	124	(81.6)	78	(57.8)	
Low and medium skilled occupations^b^	85	(29.6)	28	(18.4)	57	(42.2)	<0.001
**Family income (*****n*** **=** **272)**							
≥750 Euros	167	(61.4)	106	(73.6)	61	(47.7)	
<750 Euros	105	(38.6)	38	(26.4)	67	(52.3)	<0.001

Baseline assessment: 2019. n = 287. Natives—born in Portugal, Immigrants—born abroad and outside the EU.

^a^Managers; professionals; legislatives; technicians; and armed forces occupations.

^b^Personal service workers; industrial workers; unqualified workers; and students.

Most of the participants are women as the original cohort study focused on the caregivers and children in the household. No differences in the distribution of sex and age were found between immigrants and natives. However, immigrants were less educated (*p* = 0.021), and had lower monthly family income (*p* < 0.001) when compared to natives. Also, among immigrants, there was a lower proportion of people employed than natives, 64.4 vs. 82.2%, respectively (*p* = 0.001). Immigrants were more concentrated in low-skilled and medium-skilled occupations (personal service workers; industrial workers; unqualified workers) in contrast to natives, 40 vs. 18%, respectively, while natives were more concentrated in high-skilled occupations (managers; professionals; legislatives; and technicians; *p* < 0.001). To evaluate socioeconomic inequalities between immigrants and natives at the baseline, we estimated crude and adjusted odds of having lower education, not being employed, having lower income, and always using natives as reference class.

**Figure 2:**
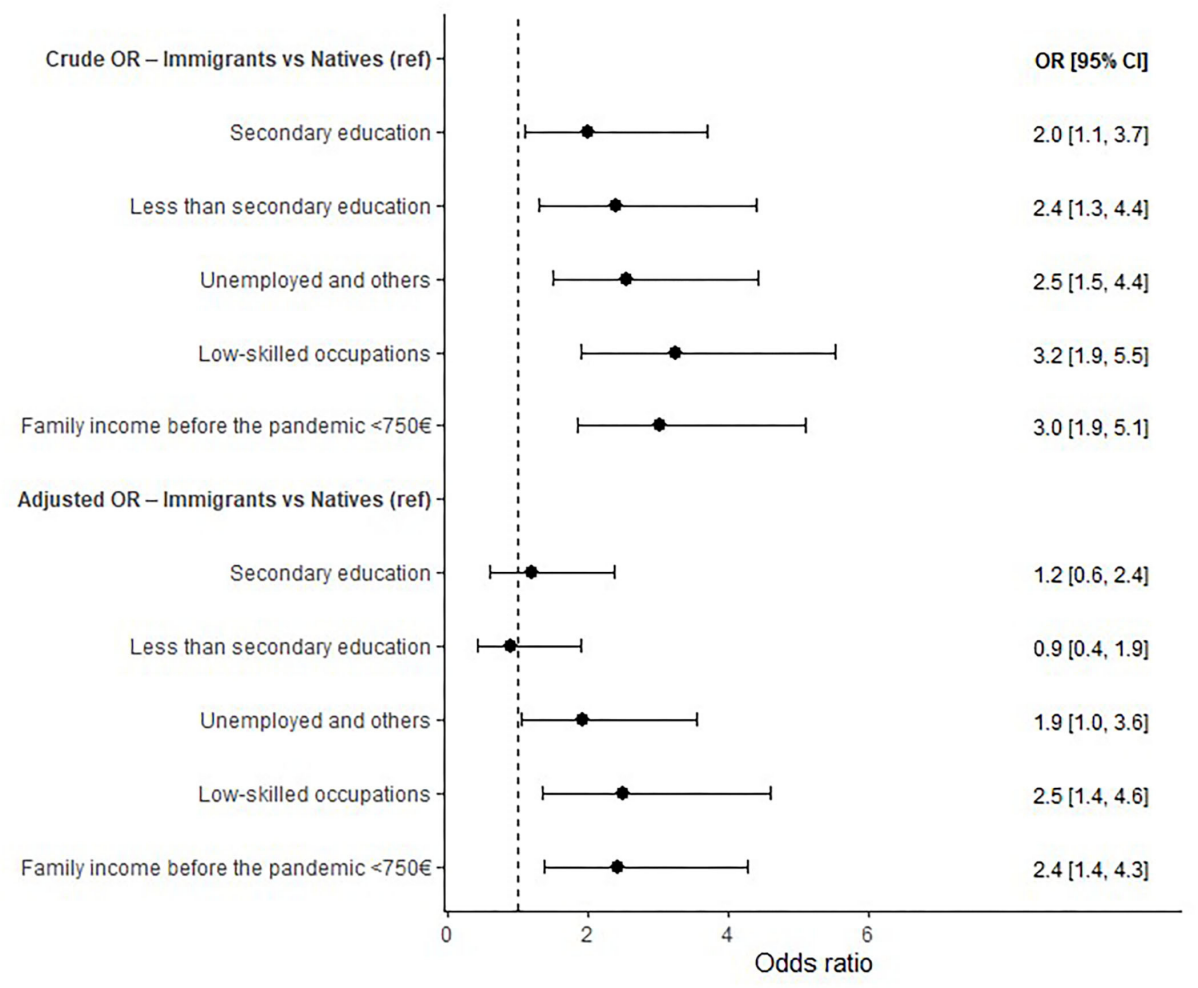
Socioeconomic inequalities between immigrants and natives before the pandemic (adjusted OR for sex and age).

As can be seen in Figure 2, before the pandemic and adjusting for other factors, immigrants when compared with natives were more likely to not be employed, to have low-skilled occupations and a lower monthly income.

Results from the July 2020 COVID-19 impact survey show that most of the changes in socioeconomic status were unfavorable to immigrants (Table 2). About 27% of them mentioned someone in the household became unemployed due to the COVID-19 pandemic compared to only 9.9% of natives (*p* < 0.001). More than 50% of immigrants were subject to temporary or partial lay-off because of COVID-19 in comparison to 32.2% lay off among natives (*p* = 0.001) and more than two-thirds of immigrants have seen their monthly household income decrease during the pandemic compared to 50% of native (*p* < 0.001).

**Table 2:** Socioeconomic impact of COVID-19 by immigrant status in July 2020, *n* = 287 (natives—born in Portugal, immigrants—born abroad and outside the EU).

	**All (*****n*** = **287)**		**Natives (*****n*** = **152)**		**Immigrants (*****n*** = **135)**		***p*-value**
	***N***	**%**	***n***	**%**	***n***	**%**	
**Someone in the household unemployed because of COVID-19 (*****n*** **=** **287)**							
No	236	(82.2)	137	(90.1)	99	(73.3)	
Yes	51	(17.8)	15	(9.9)	36	(26.7)	<0.001
**On temporary or partial on lay-off because of COVID-19 (*****n*** **=** **251)**							
No	133	(53.0)	82	(62.6)	51	(42.5)	
Yes	118	(47.0)	49	(37.4)	69	(57.5)	0.001
**Household income change (*****n*** **=** **285)**							
Increased or remained the same	112	(39.3)	75	(49.3)	37	(27.8)	
Decreased	173	(60.7)	77	(50.7)	96	(72.2)	<0.001
**Falling behind with bills (*****n*** **=** **286)**							
No	196	(68.5)	116	(76.3)	80	(59.7)	
Yes	90	(31.5)	36	(23.7)	54	(40.3)	0.003
**Financial difficulties in buying food (*****n*** **=** **286)**							
No	196	(68.5)	113	(74.3)	83	(61.9)	
Yes	90	(31.5)	39	(25.7)	51	(38.1)	0.024
**Financial difficulties in buying hygiene products (*****n*** **=** **286)**							
No	194	(67.8)	117	(77.0)	77	(57.5)	
Yes	92	(32.2)	35	(23.0)	57	(42.5)	<0.001
**Financial difficulties to pay phone and internet (*****n*** **=** **284)**							
No	187	(65.8)	121	(80.1)	66	(49.6)	
Yes	97	(34.2)	30	(19.9)	67	(50.4)	<0.001
**Kids go to school for a meal (*****n*** **=** **266)**							
No	249	(93.6)	131	(97.0)	118	(90.1)	
Yes	17	(6.4)	4	(3.0)	13	(9.9)	0.02

A higher proportion of immigrants reported financial difficulties in paying bills (40.3%), buying food (31.5%), buying hygiene products (42.5%), and paying for phone and internet (50.4%), when compared to native Portuguese, 23.7, 25.7, 23, and 19.9%, respectively, with differences being significant (*p* = 0.003; *p* = 0.024; and *p* < 0.05). About 10% of immigrants reported sending their children to school to have meals compared to 3% among natives (*p* = 0.02).

**Figure 3:**
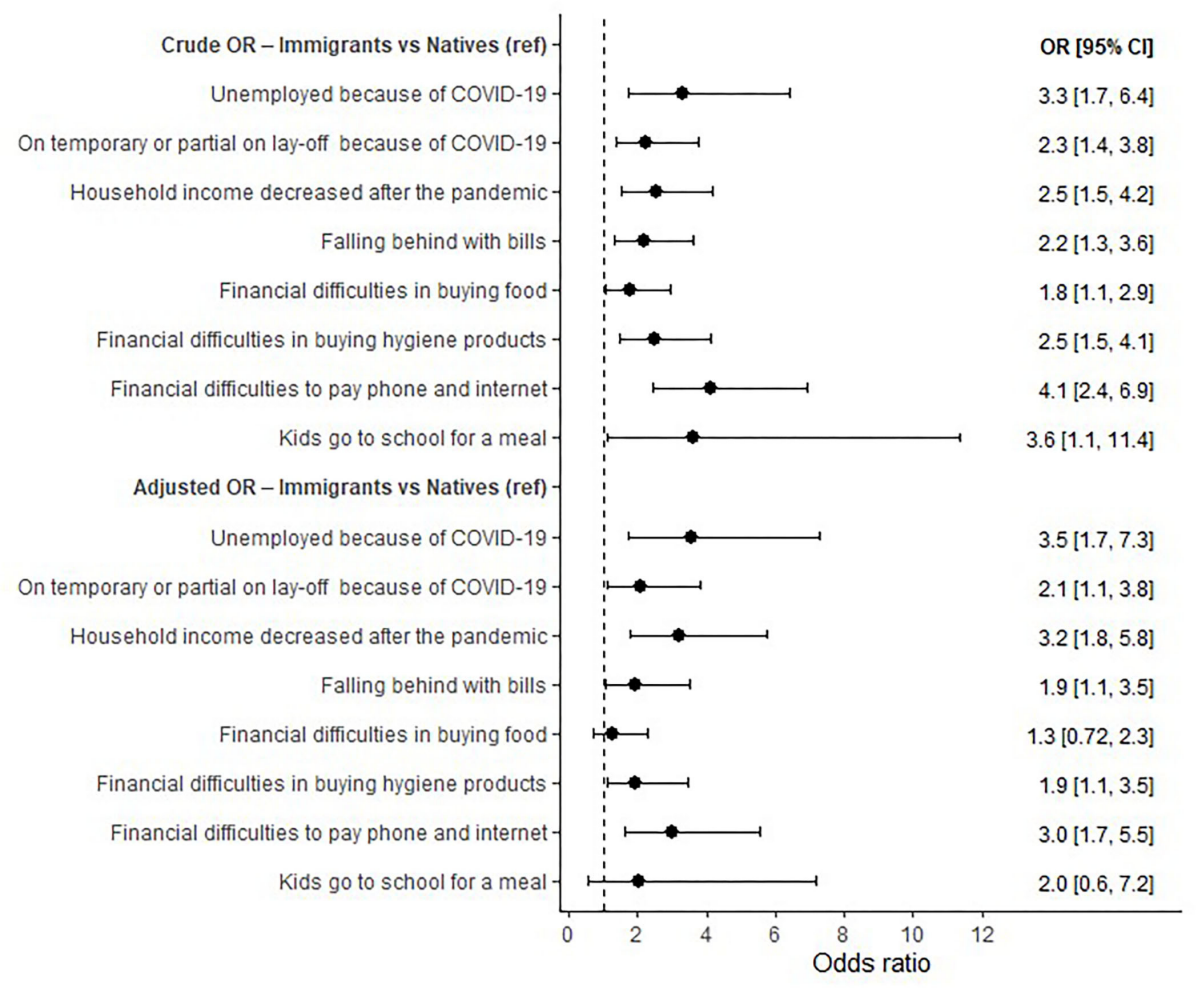
The effect of the COVID-19 pandemic on participants' economic wellbeing (immigrants vs. natives).

Figure 3 presents the effect of the COVID-19 pandemic on participants' economic wellbeing measured by crude and adjusted odds ratio taking natives as the reference class. Adjusting for sex, age, education level, occupation, and family income, immigrants were more likely to be unemployed due to the COVID-19 pandemic (AOR 3.54, 95% CI 1.72–7.30). In addition, immigrants were more likely to be subject to temporary or partial lay-offs because of COVID-19 (AOR 2.10, 95% 1.17–3.76), and to suffer a decrease in their monthly household income due to COVID-19 (AOR 3.21, 95% CI 1.80–5.75). Regarding financial difficulties during the COVID-19 pandemic, immigrants were more likely to fall behind with bills (AOR 1.95, 95% CI 1.09–3.50), to find it difficult to buy hygiene products (AOR 1.95, 95% CI 1.10–3.48), paying phone and internet bills (AOR 3.02, 95% CI 1.65–5.53).

## Discussion

This study aimed to examine the socioeconomic impact of COVID-19 on immigrants living in Amadora, Metropolitan Region of Lisbon and the extent to which preexisting inequalities had been exacerbated during the pandemic. First, analyses of variables that measure the participants' socioeconomic position before the pandemic showed preexisting socioeconomic inequalities among immigrants compared to natives. In general, socioeconomic factors have been widely used to assess health in research. For example, better socioeconomic status in terms of higher education, employment, or income may translate to better living conditions, and access to information, and hence better health outcomes (25, 26). The preexisting conditions in our study translate into immigrants being less employed, underpaid, and more likely to have low-skilled occupations compared to natives. Second, our results show disparity in the socioeconomic impact of COVID-19 among immigrants and natives, which translates into an exacerbation of immigrants' lower economic wellbeing during the pandemic. This impact is more evident in immigrants' employment status, monthly income, and financial difficulties during the pandemic. As such, our results indicate that immigrants face other types of inequality besides preexisting socioeconomic and access to health inequalities, being at higher risk to suffer adverse economic outcomes due to COVID-19. These findings are consistent with other studies that found immigrants to suffer from social and economic disparities (27, 28). Moreover, the Portuguese Migration Observatory registered several disparities among immigrants in Portugal with respect to housing, access to healthcare, low wages, and the greatest exposure to social exclusion (29).

In our study, immigrants were more likely to be unemployed, on temporary lay-off, or to have lost income due to COVID-19. These findings are consistent with findings from other studies in which immigrant workers have confronted a disproportionate social and economic impact of the pandemic (21, 30). These findings are not surprising and can be explained for several reasons. First, the previous crisis showed immigrants to be the first affected by financial crises (17, 19, 20). The global financial crisis in 2008 and the following wave in 2011, which resulted in a major recession with severe labor market depression, led to a rapid, significant increase in the immigrants' unemployment rate compared to native inhabitants (19). Second, Portugal, in 2020, has been among the most affected by any economic crisis due to COVID-19 compared to other countries (the economy contracted 16.3% in the 1st semester of 2020), given the high contribution of tourism to the Portuguese economy (31). According to the Portuguese Migration Observatory, immigrants are more likely to be employed in underpaid jobs related to the domestic services sector, construction sector, or employed in jobs linked to tourism, such as hotels, cafes, and restaurants (29). During the year 2020, about 45% of Portuguese hotels have temporarily closed or are planning to close due to the impact of the coronavirus pandemic, which kept most visitors away from our tourism-dependent country for over 9 months (32). The Portuguese Hospitality Association (AHP) estimated revenue losses of between €3.2 and €3.6 billion during 2020, and 24.8–46.4 million fewer overnight stays during the same year (33). As these sectors are among the ones strongly hitten by COVID-19 restrictions, immigrants may be the first to suffer from the economic consequences as shown in the precedent crisis, being highly represented in these sectors. For example, during the Portuguese economic recession that started in 2008 and the following financial crisis, occupations with high immigrant concentrations such as construction, accommodation, and restaurant sectors, were severely affected. Accordingly, the difference in unemployment rates between natives and immigrants had widened by 7.8% points in 2010 (34).

Our study found that immigrants living in Amadora, Lisbon Region, are more likely to face financial hardship during the pandemic, and this finding could be alarming for several reasons (35, 36). Inability to provide proper nutrition or hygiene products due to financial difficulties during the pandemic may put immigrants at higher risk of infection, especially when immigrants live in poor environmental conditions (8). In addition, over 25% of immigrants in Portugal live in overcrowded housing (29). Immigrants living in these conditions and unable to afford proper nutrition or hygiene products due to financial hardship will find themselves unable to follow essential prevention measures that include hand hygiene, social distancing, or proper self-isolation in the case of infection. Conclusions from a UK population study go in the same direction: compared to UK-born white, British, Black, Asian, and Minority Ethnic (BAME) migrants in the United Kingdom were more likely to experience job and income loss during the COVID-19 lockdown (37). Moreover, recent publications for France (38) and the United States (39) reveal an unequal burden of COVID-19 to income, race/ethnicity, and household crowding.

Historically, immigrants and ethnic minorities were among the most affected by infectious diseases during economic crises due to poor living conditions and lack of access to preventive health services and information (40, 41). All these factors can put immigrants at a higher risk of infection, especially in light of their limited ability to afford proper nutrition or hygiene products due to financial limitations (8).

To the best of our knowledge, our study is the first to capture the direct socioeconomic impact of COVID-19 among immigrants and natives in Portugal. It highlights the bidirectional relation between inequalities deeply rooted among immigrants and COVID-19. On the one hand, socioeconomic inequalities affect local patterns of COVID-19 burden, as confirmed in previous studies (2, 4, 6–8, 42, 43). On the other hand, COVID-19 affects the economic wellbeing of immigrants during the pandemic. The importance of our study lies in its ability to reveal that the current pandemic has not only exposed health disparities among immigrants but also exposed the economic vulnerability of this population during the pandemic, which is a result of long-standing structural inequalities. Moreover, our study was able to analyze preexisting socioeconomic conditions among immigrants using data from the first wave of the survey. Since the immigrant households in our study include children, special attention should be paid to the potential associated effects of this economic downturn on children, such as poor nutrition, overcrowded houses, and lack of access to technologies for online learning and during repeated lockdown periods. As Portugal ensured immigrants' access to healthcare during the pandemic, similar attention should be paid to this population's financial hardship. Inclusive policies that guarantee equal access to social security during the pandemic will be critical to protecting the immigrant population.

This study has some limitations. First, data were collected from an existing cohort study; individuals were recruited before the pandemic in a different context and the impact was measured using two sequential cross-sectional studies. However, considering the difficulty to obtain information and reaching this population during COVID-19 lockdowns, we consider they offer valuable evidence in this research area. Also, we only consider families that have at least one child. Second, we only analyzed a specific point in time (July 2020 lockdown), a period accentuated by a significant economic crisis. We implemented a similar questionnaire in January 2022, during the Omicron wave, and we believe the results will give additional and relevant insights to this study. Finally, our findings described the situation in Amadora Municipality, which cannot be assumed to represent the national reality. However, we are currently expanding the area of our study to include four other Municipalities with different socioeconomic characteristics.

## Conclusion

The COVID-19 pandemic that started as a health crisis is rapidly turning into an economic crisis, especially among immigrant populations. This study urges policymakers to take urgent actions to protect immigrants from COVID-19 adverse economic impact and guarantee social equality during this unprecedented crisis.

Our results were already presented at the regional (Regional Health Administration of Lisbon and Tagus Valley) and national level (Portuguese Parliament) and are being incorporated in public health programs, namely, in Amadora. The importance of this study was recognized at the national level, with the gold medal of the 2020 Human Rights Award of the Portuguese Parliament.

## Data availability statement

The raw data supporting the conclusions of this article will be made available by the authors, without undue reservation.

## Ethics statement

The studies involving human participants were reviewed and approved by Health Ethics Committee of the Regional Health Administration of Lisbon and Tagus Valley, Portugal (001/CES/INV/2019 and 9-2020/CES/2020). The patients/participants provided their written informed consent to participate in this study.

## Author contributions

MO: conceptualization, methodology, supervision, funding, and original draft. ANS: investigation, data analysis, and original draft. AA, DV, and ACS: methodology and review. ZM and RA: data collection and analysis. SD: methodology and data analysis. IF: methodology, data analysis, data field supervision, and review. All authors contributed to the article and approved the submitted version.

## Funding

The project was funded by a grant from the Portuguese Science and Technology Foundation (FCT), RESEARCH4COVID19, reference 065, co-financed by FAMI funds reference PT/2018/FAMI/350, and by Saúde Global e Medicina Tropical, Instituto de Higiene e Medicina Tropical, Universidade NOVA de Lisboa, Portugal, ref. UID/04413/2020.

## Conflict of interest

The authors declare that the research was conducted in the absence of any commercial or financial relationships that could be construed as a potential conflict of interest.

The reviewers NM and DL declared a shared affiliation with the author(s) MO, ANS, AA, ZM, RA, and IF to the handling editor at the time of review.

## Publisher's note

All claims expressed in this article are solely those of the authors and do not necessarily represent those of their affiliated organizations, or those of the publisher, the editors and the reviewers. Any product that may be evaluated in this article, or claim that may be made by its manufacturer, is not guaranteed or endorsed by the publisher.
